# Neurofeedback as a nonpharmacological treatment for adults with attention-deficit/hyperactivity disorder (ADHD): study protocol for a randomized controlled trial

**DOI:** 10.1186/s13063-015-0683-4

**Published:** 2015-04-18

**Authors:** Kerstin Mayer, Sarah Nicole Wyckoff, Andreas J Fallgatter, Ann-Christine Ehlis, Ute Strehl

**Affiliations:** Institute for Medical Psychology and Behavioral Neurobiology, University of Tübingen, Silcherstrasse 5, 72076 Tübingen, Germany; Sense Labs, Mesa, AZ, 1918 N. Higley Rd, 85205 Mesa, AZ USA; Department for Psychiatry and Psychotherapy, University of Tübingen, Osianderstr. 24, 72076 Tübingen, Germany; LEAD Graduate School, University of Tübingen, 72074 Tübingen, Germany; CIN Excellence Cluster, University of Tübingen, 72076 Tübingen, Germany

**Keywords:** Adult ADHD, Neurofeedback, SCP, NIRS, Biofeedback, Therapy

## Abstract

**Background:**

Neurofeedback has been applied effectively in various areas, especially in the treatment of children with attention-deficit/hyperactivity disorder (ADHD). This study protocol is designed to investigate the effect of slow cortical potential (SCP) feedback and a new form of neurofeedback using near-infrared spectroscopy (NIRS) on symptomatology and neurophysiological parameters in an adult ADHD population. A comparison of SCP and NIRS feedback therapy methods has not been previously conducted and may yield valuable findings about alternative treatments for adult ADHD.

**Methods/Design:**

The outcome of both neurofeedback techniques will be assessed over 30 treatment sessions and after a 6-month follow-up period, and then will be compared to a nonspecific biofeedback treatment. Furthermore, to investigate if treatment effects in this proof-of-principle study can be predicted by specific neurophysiological baseline parameters, regression models will be applied. Finally, a comparison with healthy controls will be conducted to evaluate deviant pretraining neurophysiological parameters, stability of assessment measures, and treatment outcome.

**Discussion:**

To date, an investigation and comparison of SCP and NIRS feedback training to an active control has not been conducted; therefore, we hope to gain valuable insights in effects and differences of these types of treatment for ADHD in adults.

**Trial registration:**

This study is registered with the German Registry of Clinical Trials: DRKS00006767, date of registration: 8 October 2014.

## Background

Attention-deficit/hyperactivity disorder (ADHD) is one of the most common disorders of childhood with a cumulative incidence of up to 7.5% by 19 years of age [1]. The primary symptoms of ADHD include inattentiveness, impulsivity, and hyperactivity, which persist into adulthood for a large proportion of children diagnosed with this disorder. The estimated prevalence of clinician-assessed adult ADHD in general and clinical populations is 4 to 5% and 7.5%, respectively [2,3]. Impairments in educational, occupational, neuropsychological and social functioning are observed [4]. Psychiatric comorbidities are highly prevalent in the adult ADHD population and include mood, anxiety, and substance abuse disorders [5].

Various findings indicate that ADHD has its origin in neurobiological dysfunctions including a major genetic component in etiology [6,7], specifically in relation to the regulation of the dopaminergic neurotransmission [8]. Relative to controls, ADHD adults have a significant reduction in orbitofrontal volumes in the left hemisphere [9], overall cortical gray matter reduction in prefrontal and anterior cingulum cortices [10] and volume reductions in the cerebellum [11]. On a functional level, a dysfunction of the prefrontal cortex (PFC) has been assumed to underlie many of the deficits observed in ADHD, with particular involvement of the dorsolateral prefrontal cortex (DLPFC) and cingulate areas (for example, [12]). Electroencephalography (EEG) studies comparing adult individuals with ADHD to healthy controls have identified a variety of brain activity patterns including increased theta/beta ratios [13,14], increased theta and alpha activity [15,16], and deviant activity in delta and beta frequencies [17]. However, brain activity patterns seem to be dependent on the ADHD subtype [18]. Event-related potentials (ERP) also differ between adult ADHD and healthy control populations, with adult ADHD patients showing enhanced N100 and N200 and decreased P300 activity [19,20] together with topographic differences in P100 and P200 [21] and P300 [22]. Additionally, altered error potentials indicate deficits in conflict monitoring [23]. Rockstroh *et al*. [24] observed impaired regulation of slow cortical potentials (SCP), a specific type of ERP in which slow direct current shifts reflect the excitation threshold of large cortical cell assemblies in children with attention problems. SCP shifts in the electrically negative direction reflect a reduction of the excitation threshold, whereas shifts in the electrical positive direction reflect an increase of the excitation threshold. These studies show the suitability of EEG/ERP recordings for measuring cortical dysregulation during resting and task conditions for participants with ADHD.

Looking at corresponding neuropsychological concepts, ADHD has repeatedly been associated with a disturbance of single or multiple executive functions such as selective and divided attention and working memory. Consequently, there have been various attempts to determine intermediate phenotype markers based on measures grounded in neuroscience [25-27]. In spite of considerable differences between the various intermediate phenotype models, deficits in executive functioning have been demonstrated in most of them and have repeatedly been suggested to underlie the disease (for example, [28]). Specifically, deficits in working memory, response inhibition, and cognitive response control have been identified in both ADHD participants and their unaffected relatives, and have therefore been suggested as cognitive intermediate phenotypes of ADHD [25,29,30].

A method optimally suited to assess the cortical aspect of these deficits is near-infrared spectroscopy (NIRS), an optical imaging method, which allows for noninvasive *in vivo* measurements of changes in the concentration of oxygenated (O2Hb) and deoxygenated (HHb) hemoglobin in brain tissue [31-33]. NIRS has repeatedly been cross-validated using, for example, blood-oxygen-level dependent (BOLD) and arterial spin labeling-based functional magnetic resonance imaging (fMRI) [34,35], confirming the physiological basis of the signal. In a NIRS study, poorer performance in a working memory task was observed for adult participants suffering from ADHD compared to healthy controls [36]. The tendency for more omission errors during a 2-back task was also associated with significantly reduced prefrontal activation in the ADHD group compared to healthy controls. Using another executive control task, prefrontal deficits in adult ADHD participants were again observed, even when no overt performance differences occurred [37]. These studies show the suitability of NIRS in measuring prefrontal control deficits during tasks of executive function in participants with ADHD.

In neurofeedback treatment, participants learn to regulate their own brain activity (for example, SCP, EEG oscillations, O_2_Hb concentration, *etcetera*) using online feedback. The training is based on an operant conditioning process in which only the desired brain activity is rewarded. As stated previously, Rockstroh *et al*. [24] observed impaired regulation of SCP and reduced negativities in anticipation of a task in children with attentional problems. Helps *et al*. [38] found that ADHD children exhibited decreased resting state very low frequency power (0.1 Hz) and attenuated power during rest-task transitions that varied significantly from healthy controls. Attenuation of power was negatively correlated with task performance, whereby participants who attenuated least made more errors, had greater variability, and slower reaction times (RT). These findings support the conceptualization of ADHD symptoms as impaired excitation threshold regulation, characterized by decreased cortical negativity. Therefore, it was hypothesized that training ADHD participants to augment negative SCP would increase the capacity to produce cortical activation necessary for concentration and cognitive tasks.

The use of SCP as a treatment parameter for children with ADHD has yielded significant reduction in ADHD symptoms, improved attention [39-41], and has produced changes in ERP, most importantly for SCP feedback of the contingent negative variation (CNV) (for example, [40,42]). A recent study by Studer et al. [43] with healthy adult participants found increased CNV amplitude after SCP feedback training. Examination of EEG during SCP-treatment indicated that children with ADHD are able to control SCPs and this skill was observed to be stable two years after the end of treatment [44]. Therefore, one of the potential advantages of neurofeedback therapy over pharmacological treatment strategies might be the stability of improvements beyond the intervention period. As this has been shown to be an effective treatment for children, we would like to investigate whether SCP feedback is an efficient treatment for adult ADHD participants as well.

In recent years, some efforts have been made to extend neurofeedback applications from the electrophysiological to a broader neuroimaging domain. Functional imaging methods such as fMRI have the advantage of a superior spatial resolution (compared to EEG), theoretically allowing for neurofeedback training of circumscribed brain areas. Investigation of fMRI feedback has subsequently shown that healthy adult subjects are able to quickly learn to self-regulate brain activity on the basis of the BOLD signal [45-47]. As NIRS and fMRI both measure the regulation of the blood metabolism, we expect similar findings with NIRS feedback. As NIRS is relatively insensitive to motion artifacts and allows measurements to be performed in a natural, relaxed sitting position, it might provide an interesting alternative to fMRI for feedback training in psychiatric disorders such as ADHD. To date, only a few studies have attempted to use functional near-infrared spectroscopy (fNIRS) with a neurofeedback protocol [48]. In a pilot project, real-time feedback was provided to train subjects to increase their motor cortex activation using motor imagery. It was shown that the signal-to-noise ratio of the O_2_Hb signal increased significantly over the course of the feedback training within five sessions. In another study, 21 subjects performed motor imagery with relevant cortical feedback and sham feedback. Only in the “verum” feedback condition has greater activation of the contralateral premotor cortex been induced [49].

Based on this preliminary work, we want to investigate the efficacy and long-term stability of fNIRS feedback in comparison with EEG feedback.

Additionally, neurophysiological predictors of treatment outcome will be investigated. Target parameters of the baseline NIRS and EEG assessments will be used as predictors in regression analyses. Based on previous findings regarding the prediction of treatment outcome via EEG markers of frontal lobe function in schizophrenic patients [50], we expect treatment effects to be particularly pronounced in participants with weak activation in the baseline frontal lobe assessments, especially for the NIRS feedback arm that is specifically focused on strengthening frontal lobe function. Regarding the EEG baseline data, mean relative theta and relative beta power as well as theta/beta ratios will be calculated for frontal and central regions of interest (ROI) during the eyes open and eyes closed resting state conditions. Additionally, P300 and CNV amplitudes and latencies will be calculated during neuropsychological assessment. Research indicates that adult ADHD participants possess elevated theta/beta ratios [13,14], which have been found to discriminate ADHD participants from healthy control populations [51,52]. These ratios may have prognostic value for prediction of outcome after stimulant medication as well as after neurofeedback treatment [18]. Elevated theta/beta ratios are hypothesized to reflect task-related brain “activation” and serve as a discriminant of behavioral performance [53]. Dominant slow wave activity has been linked with reduced auditory oddball P300 activity [54] and stimulant medication response [52], while larger pre-training CNV activation has been linked with larger symptom reductions following SCP feedback [55]. Therefore, we expect effects to be particularly pronounced in participants with elevated theta/beta ratios and decreased P300 amplitude but greater CNV negativity at baseline assessment.

## Methods/Design

In this proof-of-principle study, a total of 60 adult participants diagnosed with ADHD (combined, hyperactive, or inattentive type) are randomly blockwise assigned to SCP, NIRS, or electromyogram (EMG) feedback groups (n = 20 each) stratified for age, sex, and educational level. This is the first study to investigate and compare the efficacy of these treatment modalities in an adult ADHD population.

Twenty healthy controls matched for age, sex, IQ, and pre- and post-treatment duration will be included in the neurophysiological assessments for pre- and post-treatment measurement in order to establish “expected” activation patterns and allow for a more thorough interpretation of changes following the neurofeedback treatment. The study objectives are as follows:

1. Assess whether there are physiological differences (EEG and ERP) in ADHD participants compared to healthy controls.
2. Assess whether adult participants are able to demonstrate learning of cortical self-regulation.
3. Assess whether treatment leads to an improvement in cognition (that is, attention and working memory) and behavior (that is, restlessness and impulsivity), and changes in specific EEG or NIRS parameters.
4. Assess whether specific methods and protocols are more efficacious in changing behavioral, cognitive, and EEG and NIRS outcome variables.
5. Assess whether changes are stable at the 6-month follow-up.
6. Assess whether specific methods and protocols differ in the stability of cortical self-regulation and clinical effects at the 6-month follow-up.
7. Assess whether the efficiency of the different training protocols differs depending on individual neurocognitive baseline parameters (for example, frontal lobe activation during neuropsychological assessments; baseline CNV etc.).

### Study flow

At baseline/pre-treatment, participants are screened and diagnosed with ADHD, assessed with a battery of neuropsychological questionnaires for evaluation of cognitive and behavioral outcome variables, and evaluated using a 22-channel NeXus-32 (Mind Media B.V. with Biotrace^+^ Software, Herten, The Netherlands) continuous EEG recording (eyes closed, eyes open, auditory oddball, and auditory Go/NoGo task). Moreover, concentration changes of O_2_Hb and HHb during three executive functioning tasks (working memory, Go/NoGo, and word fluency) are measured by a 52-channel continuous wave NIRS-system (ETG-4000, Hitachi Medical Co., Tokyo, Japan). At mid-treatment (that is, after 15 sessions), post-treatment (after 30 sessions), and 6-month follow-up, the neuropsychological assessments are repeated.

**Figure 1:**
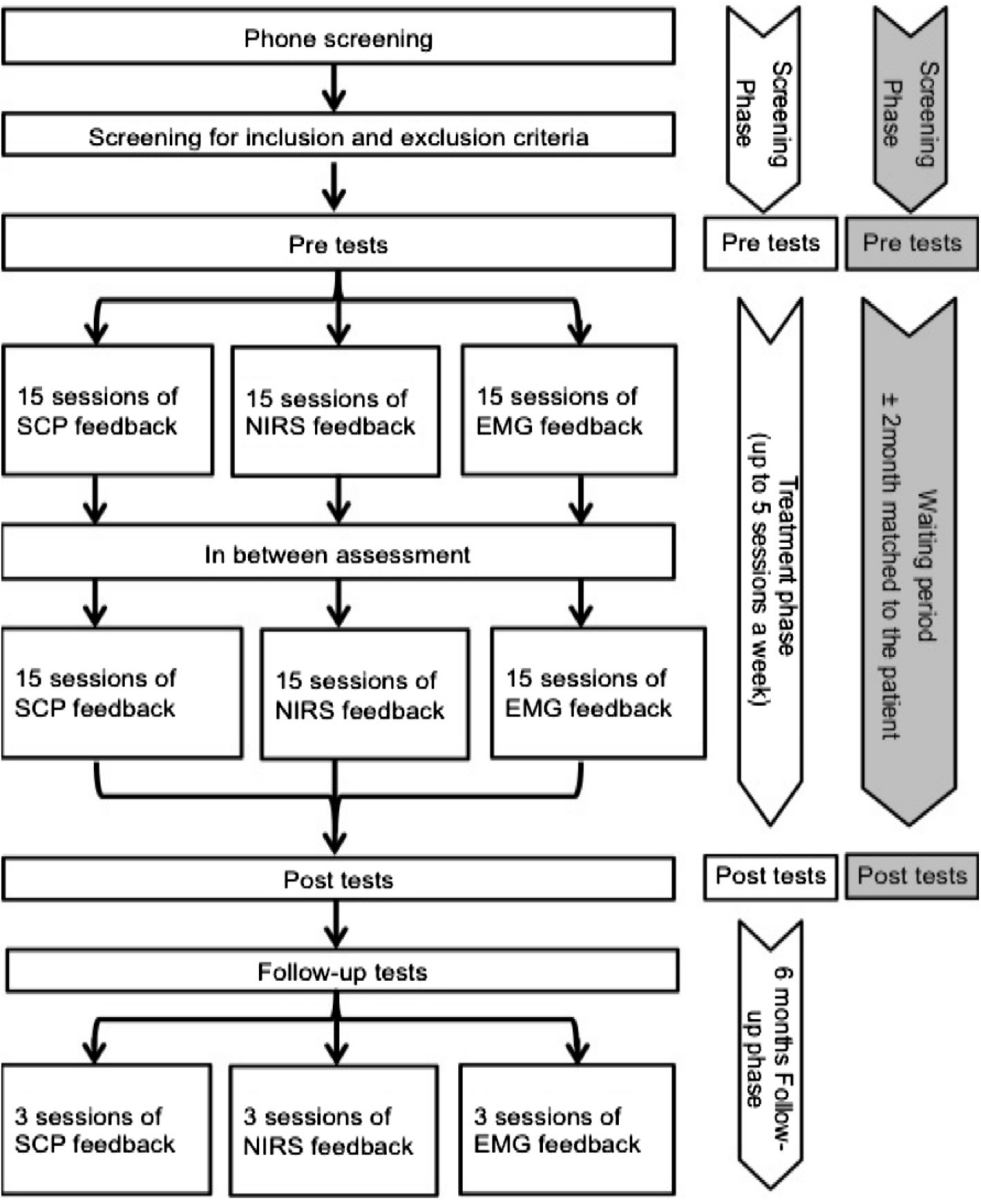
Study flow of assessments and treatment for the attention-deficit hyperactivity disorder (ADHD) groups (in white) and the control group (in gray).

Healthy controls (n = 20) undergo the EEG/NIRS assessment for the pre- and post-treatment measurement points. See Figure 1 for an illustrated overview of the study flow.

### Participants and recruitment

Participants of the ADHD group, as well as the healthy control group, are being recruited from the University of Tübingen student population, and non-student adults are being recruited through university mailing lists and by flyer. We are including all occupations and academic levels to have a diverse sample. The initial screening is conducted via phone and questionnaires are being mailed to check for the inclusion criteria. With the mailed questionnaires, potential participants also receive detailed information material and the informed consent form. The ADHD diagnostic assessment is scheduled if inclusion criteria are met. The control participants are being selected for age and sex and are included after the IQ test reveals them as a match for a patient.

### Ethics and written consent

This study (DRKS00006767) was approved by the local Ethics Committee (Ethics Committee for the Medical Department, University of Tübingen, Germany, Ethics votum number: 434/2010B01; Date 15 October 2012) according to the Declaration of Helsinki. Before entering the study, participants are informed about the study objectives, study design, and potential risks by one of the main investigators and are given this information in writing. Written consent is obtained from all participants.

Study-related information and informed consent is administered by the investigator(s) to ensure that participant questions and concerns are addressed. Participants receive the following information designed to educate him/her extensively on the subject of the investigation: information regarding the overall study; information on the goals, methods, and procedures of the study; and the end of the study plan. Participants are informed that they have the right to discontinue their participation at any time without giving a specific reason and without penalty.

### Inclusion and exclusion criteria

Participants include adults 18 years and older who meet the following inclusion criteria (please see “Assessments” section below for an explanation of all abbreviations):

1. Attention Deficit Disorder inattentive type or hyperactive type or combined type according to DSM-IV criteria (ADHS-SB ≥18; WURS-K ≥30; WRI = adult ADHD).
2. No additional serious physical, neurological, or psychiatric disorders with the exception of moderate depression (BDI-II score <28) and personality disorders, however, antisocial (SCID-II) or borderline personality disorders (BSL-23 ≤ 47) were excluded.
3. Full scale IQ >80.

Participants are excluded based on the following:

1. Self-reported diagnosis of the following current symptoms: Serious physical illness or chronic diseases such as lung disease, heart disease, diabetes, hypertension, and rheumatic diseases; neurological disorders including Parkinson’s disease, stroke, multiple sclerosis and epilepsy; or indicated psychiatric disorders including bipolar disorder, psychosis, obsessive-compulsive disorder, chronic tics, Tourette syndrome, and suicidal behavior.
2. Previous participation in another neurofeedback study.

The initial screening is conducted via phone and questionnaires are mailed to check for the inclusion criteria. The ADHD diagnostic assessment (see Table 1) will be scheduled if inclusion criteria are met. If the participants are on medications such as any short-acting stimulants, they are asked not to take them for at least 24 h prior to each assessment. For the neurofeedback treatment period, they may take their medication but on a stable dose. We do not stratify randomization for medication as we expect the usage to be very diverse.

**Table 1:** **List of all assessments and their respective time of acquisition**

**Visit**	**Screening**	**Pre-test**	**Treatment phase 1**	**In between test**	**Treatment phase 2**	**Post-test**	**FU test**	**FU treatment**
**Assessment**								
ADHD-SB	X			X		X	X	
BDI-II	X			X		X	X	
BSL-23	X			X		X	X	
STAI	X			X		X	X	
CFT-20-R	X					X		
d2-R	X					X		
WRI	X					X	X	
SCID	X							
FERT			X		X			X
Checklist			X		X			X
WURS-K	X							
Sleep Q		X		X		X	X	
PANAS EHI	X	X		X		X	X	
PPI	X							
FEA-FFB	X							
FEA-AFB	X			X		X	X	
Medical Q	X							
EEG		X		X		X	X	
NIRS		X		X		X	X	

ADHD-SB, ADHS-Selbstbeurteilungsskala (German ADHD self-rating scale); BDI-II, Beck’s Depression Inventory; BSL-23, Borderline Symptom List; STAI, State Trait Anxiety Inventory; CFT-20-R, Culture Fair Test-20 Revised; d2-R, Test of Attention; FEA-FFB and FEA-AFB, Fragebogen zur Erfassung von ADHS im Erwachsenenalter, frühere/aktuelle Probleme - Fremdbeurteilung (German ADHD third-party rating scale); FERT, Fragebogen zur Erfassung relevanter Therapiebedingungen (German therapy effect rating scale); FU, Follw-up; SCID, Structured Clinical Interview for DSM-IV Disorders; WRI, Wender-Reimherr Interview; WURS-K, Wender Utah Rating Scale; PANAS, Positive Affect Negative Affect Schedule; EHI, Edinburgh Handedness Inventory; PPI, Psychopathic Personality Inventory; Q, questionnaire; EEG, electroencephalography; NIRS, near-infrared spectroscopy.

### Interventions

#### Slow cortical potential feedback

**Figure 2:**
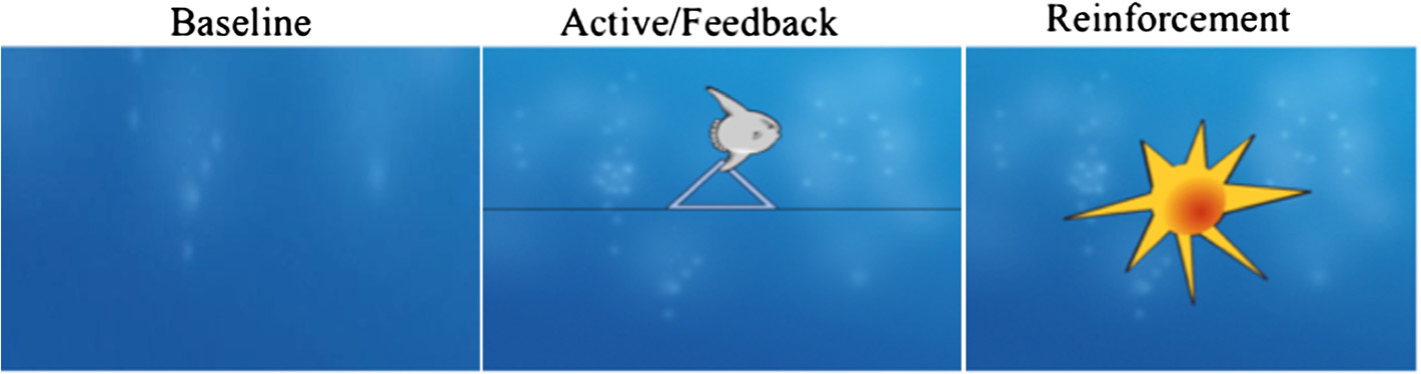
Neurofeedback trial set-up: 2sec baseline, 8sec active phase and 2sec reinforcement phase.

SCP feedback is conducted with the THERAPRAX (neuroConn GmbH, Ilmenau, Germany). The feedback protocol was developed by researchers at the Institute for Medical Psychology and Behavioral Neurobiology and has been used for many years in a variety of studies [56]. SCP are recorded at Cz referenced against mastoid A1 with a ground electrode on mastoid A2. Each SCP session consists of four runs of 40 trials, with each trial lasting 8 s and consisting of three phases: a baseline phase (seconds 0 to 2), an active phase (seconds 2 to 10), and a reinforcement phase (seconds 10 to 12) (See Figure 2). At the end of the baseline phase, participants are cued by a triangle directed to the top of the screen to “activate” their brain and by a triangle directed to the bottom of the screen to “deactivate” their brain. “Activation” in the SCP group means to produce an SCP shift in the electrically negative direction. “Deactivation” means to produce an SCP shift in the electrically positive direction. In all sessions, trials with required activation and deactivation will be randomly distributed and make up 50% of all trials.

Participants are trained one to three times per week (maximum of five times per week) for a total of 30 sessions. Each session lasts about an hour, including the preparation time and is divided into four 8-min blocks. To generalize newly acquired regulation skills to everyday life situations, the third block in each session serves as “transfer block” in which no visual feedback is presented during the active feedback phase of each trial. The level of success is indicated with the visual reward system only. Participants are instructed to use their self-regulation in everyday life situations. After the 15th session, participants are provided with a 15 × 5 inch picture of the neurofeedback screen as a memory aid as well as a CD playing a video of the transfer trials without feedback to help the transfer to daily life.

#### Near-infrared spectroscopy feedback

The extent of prefrontal activation in terms of changes in O_2_Hb concentration is displayed online on a computer screen. Based on previous fMRI feedback protocols and the SCP-feedback screen described above, participants are instructed via visual commands (triangle on the screen) to regulate PFC activity up (“activation period”) or down (“deactivation period”). During the activation period, upregulation of PFC activity is operationalized as an increase in the concentration of O_2_Hb within a predefined region of interest (NIRS channels covering left and right lateral prefrontal areas). During the deactivation period, on the other hand, participants should achieve a decrease in O_2_Hb concentration within the same target region. Functionally, such a decrease in prefrontal oxygenation below baseline levels will probably be associated with an increased blood supply in other parts of the brain, for example, visual association areas or parts of the default network. In between each “active” period (activation versus deactivation), rest trials ensure a return of hemodynamic activation to baseline levels. One session consists of a total of 24 activation periods (30 s each), separated by 30-s resting periods. Also in accordance with the SCP feedback protocol (see above), the ratio of “activation” to “deactivation periods” is 50/50 over the course of all sessions. Moreover, a block of eight “transfer trials” will be included after the first block of 12 activation trials. Participants will be trained one to three times per week for a total of 30 sessions. Each session lasts about 40 minutes, including preparation time.

#### Electromyogram feedback

For the EMG feedback, an EMG biofeedback application is used based on the protocol being already used in the above-mentioned clinical study “Neurofeedback in children with ADHD” (Ho 2503 4/1) [57]. EMG electrodes are placed over the right and left musculus supraspinatus. In addition, EEG electrodes are placed onto the scalp to imitate an EEG or NIRS recording. The relation between relaxation on the left and tension of the right muscle is used as the feedback signal when participants are asked to regulate the signal up. To regulate it down, the left muscle has to become tense while the right has to be relaxed. Trial length, visual output, transfer trials and overall duration resemble the SCP neurofeedback as described above as closely as possible.

Preliminary data from the multicenter controlled randomized study for children with ADHD [57] show superior outcome for SCP compared to EMG. In another pilot study, children with ADHD underwent NIRS feedback with comparable outcome after 12 sessions to SCP feedback [58]. This is the first study to investigate adults, and we use an identical design to rule out nonspecific effects. We compare two neurofeedback techniques (control of metabolic and electrophysiological parameters) in their effectiveness. In a second step we try to rule out nonspecific effects by using a placebo-controlled training (EMG). Expectancy effects are assessed with a questionnaire (FERT - see below) and will be included in the analysis.

### Control group

The healthy controls undergo only part of the diagnostic procedure, including the ADHD-SB, BDI, STAI, EHI, BSL, d2, CFT-20-R (see section below for explanation), demographic and medical history questionnaires, and the EEG and NIRS assessment. The controls are assessed at two time points but do not receive any treatment in between. The first measurement is being performed directly after the recruitment and screening procedure. The time between the first and the second measurement is dependent on the amount of time it takes the matched patient to complete treatment (+/− two months).

### Randomization and blinding

The randomization is conducted in two steps: first a blockwise randomization, and second a pairwise randomization for age, sex and IQ. We determined a grouping order (EEG = 1; NIRS = 2; and EMG = 3). If the participant does not fit into group 1, he/she is matched to group 2 and if not group 2, then group 3. For the next participant, the matching process starts in group 2 and so forth.

The participants are not blinded to the treatment condition, as it is easier to achieve physiological self-control knowing which parameter has to be controlled. Further, blinding was not possible, due to the different feedback methods that made it obvious which treatment was being applied and received (for example, NIRS vs. EEG measurement setup). The different methods are used in different labs and with different technical requirements, and therefore, neither blinding of participants nor blinding of assessors was possible.

### Assessments

#### Psychometric assessments

##### Medical history questionnaire (screening)

Participants are asked to indicate the following: sex; age; handedness; years of education; occupation; previous episodes of head injury with loss of consciousness; current medication and dosage history; other substances currently being taken; prior experience with EEG/NIRS; for female participants, pregnancy and oral contraceptive information. This questionnaire is used to identify inclusion and exclusion criteria.

##### ADHS-Selbstbeurteilungsskala (ADHS-SB; screening and evaluation of outcome)

The ADHS-SB is a 22-item sub-scale questionnaire of the “Homburger ADHS-Skalen für Erwachsene” (HASE, [59]). The self-report questionnaire assesses the current ADHD symptoms (according to the 18 diagnostic criteria for ADHD listed in the DSM-IV and ICD-10-R) on a 0 to 3 Likert-Scale. This questionnaire is used to identify inclusion criteria and treatment effects on ADHD symptoms over time.

##### Beck’s Depression Inventory (BDI-II)

The BDI-II is a 10-minute self-reported questionnaire assessing depression symptoms during the previous two weeks [60]. This questionnaire is used to determine inclusion and treatment effects on comorbid symptoms of depression over time.

##### Borderline Symptom Liste - Kurzform (BSL-23)

The BSL-23 is a 23-item self-report questionnaire used to assess borderline personality disorder symptoms on a 0 to 4 Likert-Scale [61]. This questionnaire is used to determine inclusion and treatment effects on comorbid symptoms of borderline over time.

##### Culture Fair Test-20 Revised (CFT-20-R; screening and follow-up)

The CFT-20-R is a nonverbal intelligence test [62]. This test is used to determine inclusion and treatment effects over time.

##### Edinburgh Handedness Inventory (EHI; screening)

The Edinburgh Handedness Inventory is a self-rated questionnaire that assesses right or left hand dominance for ten activities [63]. This inventory is used for assessments.

##### Fragebogen zur Erfassung von ADHS im Erwachsenenalter, aktuelle Probleme/frühere Probleme - Fremdbeurteilung (FEA-AFB, FEA-FFB; screening and evaluation of outcome)

The FEA-AFB and FEA-FFB are third-party questionnaires designed to evaluate current (FEA-AFB) as well as childhood (FEA-FFB) ADHD-related problems and symptoms for adult participants completed by a spouse, family member, close friend or employer [64]. These questionnaires are used to determine inclusion and treatment effects on third-party rated ADHD symptoms over time.

##### Fragebogen zur Erfassung relevanter Therapiebedingungen (FERT; evaluation of outcome)

The FERT is a self-rated questionnaire to assess relevant treatment conditions, patient expectations, and patient-therapist interactions [65]. This questionnaire is used to assess treatment expectation as a nonspecific variable.

##### Structured Clinical Interview for DSM-IV Disorders (SCID-I; SCID-II; screening)

The SCID is a semi-structured interview for the assessment of DSM-IV disorders [66]. This interview is used for inclusion and exclusion criteria.

##### Test d2- Revision Aufmerksamkeits- und Konzentrationsstest (d2-R; screening and evaluation of outcome)

The d2-R is a 10-minute assessment in which participants cross out the target letter from a series of similar letters [67]. The total number of items processed minus errors indicates combined speed and accuracy scores for attentional and inhibitory control. This test is used to assess attention in comparison with healthy controls and for treatment effects over time.

##### Wender-Reimherr Interview (WRI; screening and evaluation of outcome)

The WRI is the structured interview of the sub-scale of the HASE [59]. The interview investigates psychopathological characteristics of adult ADHD. Responses are rated on a 0 to 2 Likert-Scale. This interview is used to determine inclusion and treatment effects on third-party rated ADHD symptoms over time.

##### Wender Utah Rating Scale-Kurzform (WURS-K; screening)

The WURS-K is a 25-item sub-scale questionnaire of the HASE [59]. The questionnaire establishes a retrospective diagnosis of childhood ADHD symptoms for adult ADHD evaluation using a 0 to 4 Likert scale. This questionnaire is used to identify inclusion criteria.

##### Psychopathic Personality Inventory (PPI)

The PPI-R is a 26-item self-report measure of emotional detachment using a 1 to 4 Likert-Scale [68]. This inventory is used to identify inclusion criteria.

##### Schlaffragebogen A

The “Schlaffragebogen A” is a 23-item self-rated questionnaire that assesses sleep quality and behavior for the previous night of sleep [69]. This questionnaire is used to assess the quality of sleep before the EEG measurements.

##### State-Trait Anxiety Inventory (STAI)

The STAI is a self-rated 40-item questionnaire about temporary and long-term anxiety with a range of four possible responses to each [70]. This questionnaire is used to identify inclusion criteria and treatment effects on comorbid symptoms of anxiety over time.

##### Change of living conditions checklist

Every fifth feedback session, the participants fill in a general checklist about living conditions including changes in sleep, mood, coffee, nicotine or drug intake and any major changes in their lives. This checklist is to identify any major changes over the course of training that might lead to nonspecific treatment effects.

### Neurophysiological parameters

All neurophysiological parameters are assessed for comparison to the healthy control group and to assess changes over the course of treatment.

Quantitative EEG (QEEG) data are collected using 22 EEG channels positioned according to the international 10–20 system. Two channels of the NeXus-32 (Mind Media B.V. with Biotrace^+^ Software, Herten, The Netherlands) will be dedicated to detecting horizontal eye movements and are attached 1.5 cm lateral to the outer canthus of each eye. Two additional electrodes will be used to detect vertical eye movements and are attached 3 mm above the middle of the left eyebrow and 1.5 cm below the middle of the left lower eyelid. Study tasks include an assessment of eyes closed and eyes open resting state, auditory oddball, and auditory Go-NoGo task. The resting state assessment will consist of an 8-min alternating eye open (1 min)/eyes closed (1 min) asymmetry task, 15-min eyes-closed recording, directly followed by a 5-min eyes-open recording. We will analyze absolute and relative power for the delta (1.5-3.5 Hz), theta (3.5-7.5 Hz), alpha (7.5-12.5 Hz), and beta (12.5-25 Hz) frequency bands and calculate asymmetry, coherence, alpha peak frequency, EEG phenotype, and EEG vigilance stage changes. The variables will be used to assess differences between the adult ADHD and health control participants at baseline, as well as the pre- and post-training effects in the ADHD participants.

### Auditory P300 task (Go/NoGo-oddball task)

An auditory oddball task with three different tones (see [71]) is used to elicit P3a and P3b components to investigate inter alia attentional resources for stimulus evaluation. Participants have to press a response button whenever they hear the previously defined target tone (N = 60, 1500 Hz) in a 10.5-min paradigm with additional standard tone (N = 359, 1000 Hz) and a less frequent distractor tone (N = 60, white noise). The three stimuli are presented in a pseudo-randomized order (presentation time: 50 ms; interstimulus-interval: 1300 ms). P300 amplitude and latency over central, frontal and parietal electrodes will be calculated, as well as RT, RT variability and error rates.

### Auditory P300 task (oddball task)

An auditory oddball task with two different tones is used to elicit the P300 component. Participants have to silently count the deviant tone with their eyes closed. An 8.5 min paradigm with standard tone (N = 150, 1000 Hz) and a deviant tone (N = 49, 1500 Hz) will be used. The two stimuli are presented in a pseudo-randomized order (presentation time: 50 ms; interstimulus-interval: 1300 ms). At the end of the paradigm, the experimenter asks for the count and takes note. P300 amplitude and latency over central, frontal and parietal electrodes will be calculated.

### Auditory Go/NoGo task

An auditory Go/NoGo task with three different tones is used to assess cognitive preparation, attention, and impulsivity by instructing participants to press a response button whenever they hear a previously defined target stimulus (S2_target_, N = 50, 2000 Hz) that follows a warning stimulus (S1, N = 200, 500 Hz) at a variable presentation interval within a 14-min paradigm. All three stimuli are presented in S1-S2 or S1-S2_target_ segments. Each tone is presented for 50 ms; the duration of each segment lasts 1800 ms. The segments are presented in a pseudo-randomized order with an interstimulus-interval of 2000 to 2400 ms. The CNV amplitude over central and frontal electrodes will be calculated as well as the RT, RT variability and error rate.

### Measures of peripheral physiology

Electrocardiogram (ECG), skin conductance level (SCL) and respiration measures are simultaneously and continuously recorded using the Nexus 32 system (Mind Media B. V., Herten, The Netherlands). ECG electrodes are placed on the participants’ chest (reference/ground below right and left clavicle and the active electrode below left-side ribcage) and used to assess heart rate and heart rate variability measures. A Velcro-elastic respiration band is placed around the participants’ waist or chest, based on comfort and signal quality. Finally, the SCL sensors are fixed to the participants’ nondominant hand with Velcro-sensors. The peripheral data from heart rate, respiration and SCL will be used to assess the peripheral arousal during active (ERP tasks) and resting (eyes open/eyes closed) conditions.

### NIRS acquisition

Functional near-infrared spectroscopy (fNIRS) is conducted with the ETG-4000 Optical Topography System (Hitachi Medical Co., Tokyo, Japan), a continuous wave system working with two different wavelengths (695 ± 20 and 830 ± 20 nm) and a temporal resolution of 10 Hz. Relative changes of absorbed near-infrared light are transformed into concentration changes of O_2_Hb and HHb by means of a modified Beer-Lambert law. Two optode sets (each consisting of eight emitters and seven detectors; interoptode distance: 30 mm) are placed over left and right prefrontal regions (6 x 12 cm each). They are orientated according to the standard EEG positions F3, T3 and F4, and T4 [72,73].

### Working memory task (n-back task)

A letter *n*-back task (see, for example, [36]) with three different conditions is used to investigate functions of working memory (WM). In the 0-back condition (marginal WM load), participants have to press a response button whenever a previously defined target letter appears in a stream of different letters. In the 1-back condition (low WM load), participants have to press the response button whenever the letter appearing on the computer screen is identical to the preceding letter. In the 2-back condition (high WM load), participants have to press the response button whenever the current letter is identical to the letter presented two trials before. These three conditions are performed alternately in a blockwise fashion, separated by 30-s resting segments during which participants are instructed to sit still and relax. Letters are presented in pseudo-randomized order with a presentation time of 300 ms and an interstimulus-interval of 1700 ms, resulting in 30-s task segments. Each of the three task conditions is conducted three times; that is, participants perform nine task segments. Additionally, a 10-s baseline period precedes the first task segment. For all three conditions, a total of 12 target trials appear across task segments. The button presses are recorded, and the number of errors and correct responses, as well as the RT, will be analyzed as behavioral data. The task lasts for about 10 min.

### Response inhibition task (Go-NoGo task)

A Go-NoGo task is used to investigate executive functions in terms of response inhibition processes (see [74]). The task is designed in a block-wise fashion, comprising four Go and four NoGo blocks with a duration of 30 s each. These two blocks are presented in an ABABABAB design. In each block, a stream of 16 letters is presented (stimulus presentation time: 175 ms; interstimulus interval: 1700 ms). Participants are instructed to respond with their dominant hand via button press to each letter, but to withhold their response when the letter ‘N’ is presented. While in the Go blocks no ‘Ns’ are shown, 50% of the letters in the NoGo blocks are ‘Ns’. RTs and errors are recorded, whereupon the latter are classified into commission errors (false alarms) and omission errors (missing reactions). The task lasts for about 8 min.

### Verbal fluency task

A verbal fluency task is used to investigate executive functions. In the phonological condition (letters), participants have to name as many nouns as possible that come to their mind with a previously defined first letter for 30 s. In the semantic condition (category), participants have to name as many nouns as possible that come to their mind out of a previously defined category for 30 s. In the control condition (weekdays), participants have to name the weekdays for 30 s. These three conditions are performed alternately in a blockwise fashion, separated by 30-s resting segments during which participants are instructed to sit still and relax. Each of the three task conditions is conducted three times; that is, participants perform nine task segments. Additionally, a 10-s baseline period precedes the first task segment. The task lasts for about 10 min.

### Primary and secondary endpoints

As a primary outcome, the changes in core symptoms will be assessed by the ADHS-SB (self-report) and the FEA-AFB as well as WRI (third party evaluation). Variables of secondary outcome will include cognitive factors (that is, attention, intelligence, response inhibition, and working memory) and changes in specific EEG/ERP and NIRS parameters (that is, relative and absolute power and frequency ratios, concentration changes in O_2_Hb and HHb), as well as achievement of self-regulation.

For the pre- and postevaluation, the independent variable is the treatment (SCP, NIRS, or placebo feedback). In addition, for the comparison of the neurophysiological and cognitive data the health status (healthy and ADHD participants) constitutes another independent variable.

### Risks and side effects

The medical risks of EEG and EMG recordings are few, rare, and quickly remediable. For individuals who have highly sensitive skin, there is the possibility of temporary skin irritation caused by cleaning the scalp with abrasive cleaning gel and the subsequent application of electrode gel to improve the electrical conductivity between the skin and electrode. No electrical current is applied; only electrical output is recorded from the sensors. Additionally, the EEG acquisition and neurofeedback equipment used in this investigation are certified as medical devices (class IIa. EU) for the use with human subjects.

Risks associated with EEG- or EMG-feedback treatment are also few, rare, and quickly remediable. For individuals who are highly sensitive or susceptible, neurofeedback may precipitate a headache (muscle tension induced), hot flash, or mild anxiety (performance induced). Potential problems can be minimized and averted by obtaining an appropriate medical history prior to treatment. Side effects are typically mild, transient, and quickly remediable, allowing participants to continue the treatment.

NIRS is an optical method for examining the oxygen level of cortical tissue. Light from the near-infrared spectrum (700–1000 nm wave length) can penetrate the skull of an adult head and can be absorbed by two chromophores (oxygenated hemoglobin or reduced hemoglobin). As the two types of hemoglobin differ in the amount of light they absorb, concentration changes of both types of hemoglobin in the brain tissue can be derived, and from this, information about the brain activity can be drawn [31]. An advantage of this method, especially for ADHD participants, is its relative insensitivity to movement artifacts. There are no medical risks associated with NIRS recording; whereas for the neurofeedback aspect, side-effects are expected to be similar to the ones observed for EEG neurofeedback protocols (see above). All measurements will be conducted in the presence of an experienced research assistant.

### Statistics

#### Sample-size calculation

The basic problem with determining the number of cases needed is that there are no comparable studies about SCP training in adults and even fewer studies about the influence of the blood flow regulation. For this study, the planned sample size is based on a power calculation of a meta-analysis by Arns *et al*. [75] that showed a grand mean effect size of 0.81 for improvements in inattention in children with ADHD by EEG neurofeedback training as compared to passive or semi-active (for example, EMG) placebo trainings. Assuming a pre-defined α of 0.05, a power criterion of at least 80%, and one-sided testing, such an effect size would indicate a required sample size of n = 20 per treatment group.

### Statistical analysis

#### Electroencephalogram, near-infrared spectroscopy, electromyogram and behavioral data

All dependent variables will be tested for effects within and between groups by an analysis of variance (ANOVA) (three groups x four assessment points). For the comparison of pretest-data between ADHD and healthy subjects, t-tests and/or one-way ANOVAs will be applied. If normal distribution cannot be assumed, non-parametric tests will be used.

#### Neurophysiological predictors

To investigate neurophysiological predictors of treatment outcome, target parameters of the baseline NIRS, EEG, and peripheral physiology data assessments will be analyzed in all treatment groups (SCP, NIRS, and EMG). Specific to the NIRS assessment parameters, mean task-related activation (changes in the concentration of O_2_Hb and HHb) during the baseline assessment will be individually calculated for both the 2-back condition of the working memory (n-back) task and the NoGo condition of the Go-NoGo paradigm. Specific to the EEG assessment parameters, like mean relative theta and relative beta power and theta/beta ratios will be calculated for frontal and central ROIs during eyes open and eyes closed resting state conditions. Additionally, P300 and CNV amplitudes and latencies for these ROIs will be calculated during neuropsychological assessments. The peripheral data from heart rate, respiration, and SCL will be used to assess the peripheral arousal during active (ERP tasks) and resting (eyes open/eyes closed) conditions. These parameters will then be used as independent variables in a regression analysis to predict treatment success (as indicated by changes in psychometric scores in the ADHD symptom scales).

#### Training-data

Session 1 will be discarded because it is assumed that participants still have to habituate to the setting. NIRS, SCP and EMG feedback data will be analyzed to determine:

1. If participants in treatment groups are able to learn self-regulation of the trained parameter over the course of treatment and at 6-month follow-up.
2. If the difference between activation and deactivation changes throughout treatment.
3. Further, the participants will be divided into learners and nonlearners of self-regulation for further analysis.

### Slow cortical potential group

For each participant, mean differences between SCP amplitudes during both tasks (negativity/positivity) will be calculated. The differences between SCP amplitude in activation and deactivation will be analyzed separately over the cause of treatment to assess the acquired self-regulation abilities. This will be analyzed for feedback and transfer conditions.

### Electromyogram group

Data will be analyzed in the same manner as the SCP data.

### Near-infrared spectroscopy group

Hemodynamic responses will be quantified for tasks (activation/deactivation/rest), conditions (feedback/transfer) and over the course of treatment. Differences between hemodynamic response amplitudes in activation versus deactivation (as well as rest) trials will be analyzed separately for each assessment point. Changes in the hemodynamic responses for activation and deactivation trials over time (as well as changes in the difference of activation versus deactivation trials) will be analyzed and computed for both feedback and transfer conditions.

## Discussion

This paper presents the protocol and design of a randomized controlled trial with two types of neurofeedback (SCP and NIRS) and an active control (EMG biofeedback) condition for adults with ADHD. This is the first study to systematically investigate neurofeedback in adults with ADHD. It is also the first study to compare SCP and NIRS feedback to an active control condition on the one hand and to investigate this in an adult ADHD population on the other hand.

If one or both feedback types are superior to the control condition, a first step will be made towards a new acknowledged treatment option for adult ADHD.

This is not the “gold standard” design for treatment studies, which is a placebo-controlled randomized double blind design [76]. The problems with this approach are widely discussed elsewhere [77,78]. Therefore, to allow for all therapeutic aspects of neurofeedback therapy to come into effect, such as a good patient-therapist relationship, psychoeducation, and strengthening of self-efficacy expectations, we decided to use an active control condition that was used in other studies before. The study design was based on a study protocol of a multicenter treatment study with ADHD children [57]. The use of EMG feedback as an active control condition is based on the rationale that EMG feedback is not an “empty” treatment, but will induce identical nonspecific (placebo) effects that will help to differentiate between specific effects of the neurofeedback and nonspecific effects from the above-mentioned aspects.

### Possible limitations

As this study is being conducted with adults, we did not implement transfer exercises from the study on children [57] in which the children performed homework in combination with transfer trials in the lab. We also did not include a token system in which good cooperation was rewarded with stickers that the children were able to exchange for little presents or coupons. Whether this motivational aspect should have been implemented, remains open.

The results of this study are important in several aspects. First, there is a need for more research in the field of adult ADHD and especially in the field of neurofeedback treatment for adult participants with ADHD. ADHD changes its characteristics with maturation, and therefore, findings from a childhood population have only a limited impact on adult ADHD research. Second, NIRS feedback is a new and potentially more time-efficient type of self-regulation therapy. To date, an investigation and comparison of SCP and NIRS feedback training to an active control has not been conducted; therefore, we hope to gain valuable insights in effects and differences of these types of treatment for ADHD in adults.

## Trial status

The trial is ongoing.

## Abbreviations

ADHD: Attention-deficit hyperactivity disorder
ADHD-SB: ADHS Selbstbeurteilungsskala
ANOVA: Analysis of variance
BDI-II: Beck’s depression inventory
BSL-23: Borderline symptom list
BOLD: Blood-oxygen-level dependent
CFT 20-R: Culture fair test-20 revised
CNV: Contingent negative variation
DLPFC: Dorsolateral prefrontal cortex
d2-R: Test of attention
ECG: Electrocardiogram
EEG: Electroencephalogram
EHI: Edinburgh handedness inventory
EMG: Electromyogram
ERP: Event-related potential
FEA-FFB and FEA-AFB: Fragebogen zur Erfassung von ADHS im Erwachsenenalter, frühere/aktuelle Probleme - Fremdbeurteilung (German ADHD third-party rating scale)
fMRI: Functional magnetic resonance imaging
FERT: Fragebogen zur Erfassung relevanter Therapiebedingungen (German Therapy Effect Rating Scale)
fNIRS: Functional near-infrared spectroscopy
HHb: Deoxygenated hemoglobin
NIRS: Near-infrared spectroscopy
O2Hb: Oxygenated hemoglobin
PANAS: Positive affect negative affect schedule
PFC: Prefrontal cortex
PPI: Psychopathic personality inventory
RT: Reaction time
Q: Questionnaire
ROI: Region of interest
SCID: Structured clinical interview for DSM-IV disorders
SCL: Skin conductance level
SCP: Slow cortical potential
STAI: State-trait anxiety inventory
WRI: Wender-reimherr interview
WURS-K: Wender utah rating scale
